# Public Knowledge, Attitudes, and Practices towards Antibiotic Use and Antimicrobial Resistance in Eastern Region of Bosnia and Herzegovina in the COVID-19 Pandemic

**DOI:** 10.3390/antibiotics12081274

**Published:** 2023-08-02

**Authors:** Dragana Drakul, Bojan Joksimović, Marija Milić, Milica Radanović, Nikolina Dukić, Nenad Lalović, Desmond Nischolson, Biljana Mijović, Dragana Sokolović

**Affiliations:** 1 Faculty of Medicine Foča, University of East Sarajevo, 73300 Foča, Bosnia and Herzegovina; dragana.drakul@ues.rs.ba (D.D.); bojan.joksimovic@ues.rs.ba (B.J.); milica.radanovic@ues.rs.ba (M.R.); nikolina.dukic@ues.rs.ba (N.D.); nenad.lalovic@ues.rs.ba (N.L.); biljana.mijovic@ues.rs.ba (B.M.); 2 Department of Epidemiology, Faculty of Medicine, University of Pristina Temporarily Seated in Kosovska Mitrovica, 38220 Kosovska Mitrovica, Serbia; marija.milic@med.pr.ac.rs; 3 University Hospital Foča, 73300 Foča, Bosnia and Herzegovina; 4 Department of Regional Health Services Region Five, Ministry of Public Health, Georgetown 101110, Guyana; desmondnicholson42@gmail.com

**Keywords:** antibiotics, antimicrobial resistance, antibiotics knowledge, antibiotic attitude, antibiotics practices, questionnaire, COVID-19

## Abstract

The constant worsening of antimicrobial resistance (AMR) imposes the need for an urgent response. Use of antibiotics (AB), both due to irrational prescribing by doctors and irrational use by patients, is recognized as one of the leading causes of this problem. This study aimed to identify knowledge, attitudes, and practices about AB use and AMR within the general population, stratified by age, gender, and urban/rural areas during the COVID-19 pandemic. This questionnaire-based cross-sectional study was conducted in April 2022 among patients who visited three health centers in the eastern region of Bosnia and Herzegovina. A high frequency of AB use was observed during the COVID-19 pandemic (64.2% of respondents were treated with AB). Age and place of residence have not been shown to be factors associated with AB use practices that pose a risk for AMR. However, female gender (β = 0.063; *p* = 0.041), better knowledge (β = 0.226; *p* < 0.001), and positive attitudes (β = 0.170; *p* < 0.001) about use of AB and towards to AMR proved to be factors associated with better practice by respondents. Women, younger respondents, and respondents from urban areas showed better knowledge, attitudes, and behavior about the use of AB and AMR during the COVID-19 pandemic.

## 1. Introduction

Antibiotics (AB) are one of the most effective groups of drugs that relatively quickly cure the infection by eliminating the bacterial agent. The introduction of AB had a huge positive impact on global health [1,2]. High safety in AB application without pronounced toxic effects has led to their widespread use in the general population [3]. The constant tendency of bacteria to avoid the effects of AB in the last few decades has resulted in the development of multiresistant (MR), extensively resistant (XR), and even panresistant (PR) strains [1]. The emergence of the COVID-19 pandemic has led to increased number of hospitalized patients worldwide and to the overuse of antibiotics, resulting in further worsening of AMR [4]. In Bosnia and Herzegovina (B&H) before the COVID-19 pandemic, the consumption of AB expressed in defined daily dose per thousand inhabitants was higher than in the countries of Western Europe and this difference continued to increase during the COVID-19 pandemic [5]. Unlike many European countries where the consumption of AB decreased during the COVID-19 pandemic, an increase in the consumption of AB was observed in B&H, both in inpatient and outpatient settings [5,6]. In addition to the excessive and often unjustified use of AB in human medicine, the use of these drugs in veterinary medicine, agriculture, and food production contributes to the development and spread of resistance [7]. The burden of responsibility for the irrational use of AB lies not only on the irrational prescribing and distribution of AB or noncompliance with infection prevention measures by healthcare workers, but also in the improper use of AB in the general population. Improper use and AB self-medication were found more frequently in Eastern and Southern than Western and Northern European countries [8,9]. Current misconceptions about the beneficial effect of AB in viral infections, fever, and cough make the problem of irrational use of AB extremely complex. The belief that the use of AB leads to a faster cure at the appearance of the first symptoms of infection, along with the long-standing practice of distributing AB without a doctor’s prescription in the countries of Southeastern Europe, is a frequent phenomenon [9,10,11,12]. Most of those who take AB do not have the necessary knowledge about the dosage and adequate use of AB [2]. It has been shown that the general population in Serbia has a low level of knowledge about the proper use of AB and they are more inclined to self-medication [9]. The level of knowledge about the correct use of AB and antimicrobial resistance (AMR) in Serbia is significantly lower than in developed European countries such as Norway, Sweden, and the Netherlands [11]. AMR is a problem for which there is no easy or cheap solution and requires simultaneous changes within the health system as well as the general population.

Education of the general population can play an important role in reducing the inappropriate and excessive use of AB [13]. This study aimed to estimate knowledge, attitudes, and practices about antimicrobial use and AMR within the general population of B&H stratified by age, gender, and urban/rural areas in the COVID-19 pandemic.

## 2. Results

Our research included 1002 respondents, users of primary health care in three cities of B&H, Višegrad, Foča, and Goražde. Respondents were mostly females (56.6%), aged 16 to 44 (57.2%) from urban areas (72.1%) (Table 1). Almost 20% of respondents stated that they would stop taking AB as soon as they felt better, 32.4% of respondents knew that the use of AB in agriculture had an impact on the success of treatment with AB in humans, 63.3% heard of the term AMR, and only 20.9% heard that term from their physician. Almost 80% of respondents stated that AMR happened when the body became resistant to AB, 82.2% knew that many bacteria were becoming increasingly resistant to AB, while 79% knew that if a bacterium was resistant, it would become very difficult or almost impossible to treat the infection it caused. More than two thirds of respondents (72.8%) stated that AMR was a problem which could affect them or their families, 32.3% thought that AMR was a problem happening in other countries, but not theirs, 56.3% thought that AMR bacteria could be transmitted from person to person, and 73.8% knew that AMR infections could reduce the chances for success of applied medical procedures.

When it comes to the attitudes, the majority (92.9%) of respondents had an attitude that people should be treated with AB only when a doctor prescribed them, 85.8% stated that animals should be treated with AB only when prescribed by a veterinarian, and 85.3% thought that doctors should prescribe AB only when there was an indication for it. More than a half of respondents (60.9%) had the attitude that AMR was a problem only for those people using AB frequently and 69.6% stated that pharmaceutical companies were constantly creating new AB. The attitude that people should wash their hands regularly shared 97.2% of respondents, while 92.7% of respondents stated that parents should make sure that their children’s vaccinations were carried out regularly (Table 1).

A high frequency of AB use was observed during the COVID-19 pandemic (64.2% of respondents were treated with AB). Older respondents and women recorded a significantly higher use of AB in the previous month when compared to younger respondents (19.1 vs. 14.7%) and men (18.3 vs. 14.3%). There was no difference in the frequency of AB treatment in the previous year among the categories age, gender, and place of residence. Older respondents in comparison to younger respondents often used combinations of two (22.4% vs. 18.7%) and three or more AB (7.1% vs. 3.9%) at significantly higher rates without differences by gender and place of residence (*p* = 0.006) (Table 2). Women significantly more frequently than men stated that during the last use, AB were prescribed by the doctor (88.2% vs. 79.3%, *p* = 0.001). The difference by age and place of residence was not observed (Table 2).

When compared to older respondents (more than 44 years), younger respondents (16 to 44 years) significantly were more likely to be familiar with the term AMR (70.5% vs. 53.6%, *p* < 0.001), and they more often heard of this term from their doctors (22.7% vs. 18.4%, *p* < 0.001). Also, younger respondents significantly less often thought that resistant bacteria could be transmitted from person to person when compared with the older group of examinees (28.4% vs. 37.5%, *p* = 0.002) (Table 3).

Female respondents and those from rural areas (25%) demonstrated significantly less knowledge about duration of AB use compared to males (77.8 vs. 64.1%, *p* = 0.001) and those from urban areas (25% vs. 17.7%, *p* < 0.001). Significantly more female and respondents from urban areas heard of the term AMR when compared to male (70.4% vs. 54%, *p* < 0.001) and respondents from rural areas (67.9% vs. 51.4%, *p* < 0.001). In addition, significantly more females than males (25% vs. 15.4%, *p* < 0.001) and respondents from urban than rural areas (24.1% vs. 12.5%, *p* < 0.001) heard of the term AMR from their doctor. Women also showed better knowledge compared to men (36.3% vs. 27.4%) about the use of AB in agriculture and their impact on the success of treatment in humans (*p* = 0.006). However, the difference was not observed between respondents from different areas. Significantly more women than men (86.4% vs. 76.8%) and respondents from urban than rural areas (85.2% vs. 76.4%) shared the opinion that many bacteria were becoming increasingly resistant to AB. The awareness about the fact that it can be very difficult or almost impossible to treat the infection caused by resistant bacteria was higher among females than males (84% vs. 72.6%, *p* < 0.001). No difference in awareness was observed after stratification by age. Significantly more women than men (76% vs. 68.5%, *p* = 0.008) and respondents from urban than rural areas (75.6% vs. 65.4%, *p* = 0.001) knew that AMR was the problem that could affect them or their families. However, more men than women (36.3% vs. 29.3%, *p* = 0.018) and more respondents from rural than urban areas (40% vs. 29.4, *p* = 0.001) thought that AMR was the problem in other countries, but not in theirs. Also, more women and respondents from urban areas shared the opinion that AMR infections could reduce the success of medical procedures when compared to men (77.1% vs. 69.4%, *p* = 0.006) and respondents from rural areas (75.8% vs. 68.6%, *p* = 0.020). The differences in other aspects of knowledge between groups of respondents stratified by age, gender, or place of residence are not observed Table 3.

When it comes to the attitude that AMR is a problem only for those people who use AB frequently, more often, a significant number of older respondents shared this attitude than younger group of respondents (67.6% vs. 55.8%, *p* < 0.001). Significantly more women than men (95.4% vs. 89.7%, *p* = 0.001) and respondents from urban versus rural areas (95.2% vs. 87.1%, *p* < 0.001) shared the attitude that people should be treated with AB only when a doctor prescribed them. Significantly more women and respondents from urban areas shared the attitude that AR happened when the body became resistant to AB, when compared to men (83.1% vs. 75.6%, *p* = 0.004) and respondents from rural areas (82.1% vs. 73.9%, *p* = 0.004). Also, a higher percentage of women compared to men (94.7% vs. 95.2%, *p* = 0.015) and respondents from urban compared to rural areas (93.9% vs. 89.6%, *p* = 0.046) had the attitude that animals should be treated with AB only when prescribed by a veterinarian. While women more often than men (94.7% vs. 90.1%, *p* = 0.015) stated that parents should be sure that children’s vaccinations were carried out regularly, this difference was not observed between age categories. However, respondents from urban areas more often than those from rural areas (93.9% vs. 89.6%, *p* = 0.046) stated that parents should be sure that children’s vaccinations were carried out regularly. Also, more often, a significant number of respondents from urban areas compared to respondents from rural areas (71.9% vs. 63.6%, *p* = 0.022) had the attitude that pharmaceutical companies were constantly creating new AB. No difference between genders was observed. Neither were differences in other aspects of attitudes between groups of respondents relative to age, gender, or place of residence are observed (Table 4).

Using univariate analyses, sociodemographic variables (age, gender, and place of residence) showed significant associations with the respondents’ knowledge, attitudes, and practices regarding to use of AB (Table 2, Table 3 and Table 4). Multivariate analysis showed that age and place of residence were not significantly associated with AB use in practice. However, female gender (β = 0.063; *p* = 0.041), better knowledge (β = 0.226; *p* < 0.001), and attitudes (β = 0.170; *p* < 0.001) regarding to use of AB and towards to AMR proved to be factors associated with better practice by respondents. As can be seen, the highest beta coefficient was noticed for knowledge variable (β = 0.226), which means that this is the strongest identified of all significant factors about use of antibiotics in practice (Table 5).

## 3. Discussion

The aim of this study was to indicate the state of knowledge, attitudes, and practice toward AB use and AMR in the general population of B&H. Based on the observed results, there was a lack of knowledge and incorrect attitudes toward AB use, AMR, and physicians’ recommendations with significant differences regarding gender, age, and urban/rural place of residence among respondents. Also, incorrect behavior regarding AB use in practice was observed. As many as 14% of participants take AB without a physician’s prescription. Antibiotic self-medication in B&H was more pronounced than in Northern European countries, where it was registered in less than 3% people [9]. Similar rates of AB self-medication like in B&H were found in southern European countries such as Spain, Italy, and Malta [9], while higher rates were reported in Eastern Europe countries [8]. It is possible that the frequency of AB use without prescription which was found in our study was lower than the actual rate. Because the survey was conducted in health centers, in the vicinity of doctors’ offices, the results could be taken with care. It is possible that respondents were prone to over-report desirable behavior and not to report practices that were opposite to medical advice. The lack of knowledge was recognized as the main factor associated with the incorrect behavior regarding AB treatment. Considering the results of our research, the high level of AB consumption in B&H before the COVID-19 pandemic and the increase in AB consumption during the pandemic indicate the need for an urgent response to solve this problem.

The observed high frequency of AB use in the Gornje Podrinje region is in agreement with the registered high consumption of AB throughout B&H during the COVID-19 [5]. Unlike many Western European countries, where after the initial increase in AB consumption at the very beginning of the pandemic where there was a decrease in its consumption, in B&H, the trend of AB consumption, in both outpatient and inpatient settings, was constantly maintained [5,6,14,15]. In addition to strict adherence to the treatment guidelines for COVID-19, epidemic-control measures also contributed to a reduction in AB consumption during the pandemic [16]. High-impact population interventions leading to a reduction in AMR are increased community hygiene and vaccination. According to our results, across the general population of B&H positive attitudes toward these measures are widespread, probably due to the numerous educational campaigns that were carried out during the COVID-19 pandemic [16].

Of particular concern is the increase in the consumption of reserve groups of AB during hospital treatment, which implies an increased frequency of infections with resistant strains of bacteria and poor therapeutic success [17,18]. According to our results, the use of AB without a doctor’s prescription also contributed to the increase in AB use. Males are more prone to use AB without prescription than females. This finding is in accordance with numerous studies which found that males had a lower level of knowledge regarding AB use, more frequently consider AB as medication appropriate for viral infections and consequently had worse behavior regarding the use of AB in practice [19]. Except for a higher level of knowledge, the reason for better use of AB in females can be the fact that women visit their practitioner’s office more often [8,19]. On the other hand, despite better knowledge being observed in women than men in the Malaysian population, women were more prone to AB self-medication [8]. A downward trend in the sale of AB without prescriptions in private pharmacies has been registered in Serbia. It decreased from around 24% in 2007 to approximately 17% in 2017 [9]. According to our results, the most frequent method of obtaining AB when they were not prescribed by medical doctor was through family members, friends, as well as from the remaining own reserves. This can be explained by the low level of knowledge about the correct use of AB, especially about the adequate length of AB use. Many respondents shared the opinion that the use of AB should be stopped as soon as they feel better. This should be postulated as an important finding in future campaigns aiming to improve the knowledge of the general population on rational AB use.

Although more than 60% of respondents have heard of the term AMR, this study revealed that only about third of them understand its meaning. Male sex, older age, and rural areas were associated with less knowledge about AMR. It is in accordance with findings within the general population of Romania, where it was also found that younger and urban people received education mostly from their physicians, friends, and the internet, while older and rural people were informed mostly by pharmacists [8]. In the B&H population, the problem of AMR is not taken seriously enough, with more than 2/3 of the respondents believing that it is a problem of other countries and that it has no effect on them. Over 40% of respondents are not aware that AMR strains can be transmitted from person to person. Also, there is insufficient information about the importance of controlling the application of AB in agriculture and its connection with the development of AMR. We assume that the poor knowledge about AMR is caused by flawed ways of educating a large part of the population. Only 21% of respondents were informed about the term AMR by a doctor. It implies an urgent need for national educational campaigns about AB use and AMR which should be carried out by doctors and pharmacists [13,20]. A low level of knowledge about AB in the general population is associated with a higher frequency of self-medication and prophylactic use of AB [9,21,22].

This study revealed that there is a widespread misconception among the population of B&H, especially older respondents, and respondents from rural areas that AMR infections develop only in persons frequently treated with AB. The population in rural areas is less aware of the need for a physician to indicate the use of AB, which indicates the importance of conducting a campaign among these categories of residents. Although according to our results, we did not find a difference in the frequency of self-medication between the urban and rural population; this result should be taken with caution, bearing in mind that there are not enough family medicine clinics in rural areas in B&H. This may be an additional reason for AB self-medication in rural areas. Our results indicate a high level of public awareness of the importance of regular vaccination and hygiene measures in protection against infections. It is likely that the recent COVID-19 pandemic and numerous public campaigns on the prevention of this infection had a positive impact on these issues.

## 4. Materials and Methods

### 4.1. Study Design and Population

A cross-sectional study was conducted among adults in Gornje Podrinje. The research was conducted in three health centers (Foča, Goražde, and Višegrad) in B&H, during the April 2022. The selection of respondents was random. Adults who visited the family doctor’s clinic at the time of the survey and were not referred to hospital treatment were surveyed. Family medicine doctors were not present when completing the survey to avoid bias. The data collectors (6th year medical students) informed the respondents in detail about the purpose and design of the study. They assured the respondents of the anonymity and confidentiality of the data before starting to fill out the questionnaire. Respondents were informed that they would not receive monetary compensation for their participation in the research. A total of 1002 respondents were surveyed.

### 4.2. Questionnaire

For the research, an anonymous, independently created survey questionnaire was used, which was adapted to the examined population in accordance with the results of a pilot study conducted previously with 30 respondents. The results of the pilot survey were not included in the final statistical analysis. The questions in the survey were divided into four parts: demographic data (four questions), knowledge about AB and AMR (11 questions), attitudes about AB, questions about prevention measures against infections and AMR (seven questions), and questions about the practice of AB use during the COVID-19 pandemic (five questions). Through the first part of the survey, information was gathered about the respondent’s sex, age, place of residence (rural vs. urban), as well as the level of professional education. As part of the questions about knowledge, we examined knowledge about the correct use of AB. Within the same part, there were questions about knowledge of the AMR term, its spreading, and consequences. In the next part of the survey, the questions were designed to identify respondents’ attitudes towards the effects of AB, trust in medical recommendations on the AB use, and trust in public health recommendations. Questions about practical use should identify the frequency of AB use during COVID-19, the number of AB used and the way of obtaining AB (through a doctor, through health institutions, or bypassing the health system). Respondents were offered closed-ended statements with a possible larger choice of answers or a choice between true/false answers.

### 4.3. Ethical Approval

The study was approved by the Ethics Committee of the Faculty of Medicine (14 March 2022; number: 01-2-50). Since the survey was conducted anonymously, only verbal consent was taken from the respondents. The examinees were informed that only the aggregate results would be published.

### 4.4. Data Analysis and Statistics

The methods of descriptive and analytical statistics were used for data description and analysis. For the univariate analyses of differences in the frequencies of AB use by age, gender, and place of residence, a nonparametric Chi-square test was performed. Multiple linear regression was applied to assess factors associated with the use of AB in practice and AMR. The usual value of *p* < 0.05 was taken as the level of statistical significance of differences. All statistical analyses were performed using IBM SPSS Statistics Software version 24.0 for Windows (IBM Corp., Armonk, NY, USA).

## 5. Conclusions

These results point to the necessity of introducing educational campaigns to raise awareness of the importance of the rational use of AB in human and veterinary medicine. Educational campaigns should be carried out exclusively by health professionals, preferably doctors and pharmacists, because they will likely have a significant influence on the achievement of a greater degree of control over the use of AB and, thus, the reduction in AMR.

## 6. Limitations

It was a cross-sectional designed study which implies some uncertainty regarding the causal relationships between variables. Because family doctors were not examiners in this study, respondents’ bias was possible. It was not possible to accurately access respondent’s honesty. The pilot survey showed the need to shorten the survey to be acceptable to respondents. The survey had to be short enough to be completed in 10 min or less, so it would only encompass the pandemic period. It was not possible to compare KAP toward AB use and AMR before and during the COVID-19 pandemic. It was not possible to evaluate if there were changes in KAP under COVID-19 influence even though change in the AB consumption was well described. Also, another limitation may be the small number of respondents from rural areas compared to urban areas. Despite many respondents, this study cannot reflect the state of KAP toward AB and AMR in the whole of B&H because it was conducted only in limited area and should be taken carefully. Considering that only one similar study was conducted earlier in B&H, this study contributes to better understanding the current situation.

## Figures and Tables

**Table 1:** Sociodemographic characteristics, knowledge, and attitudes of respondents about the use of AB and AMR.

Sociodemographic Characteristics, Knowledge, and Attitudes of Respondents about the Use of AB and AMR	Total (*n* = 1002)	
	*n*	%
Socio-demographic characteristics		
Age, male		
16 do 44 years	573	57.2
45 to 72 years	429	42.8
Gender, female	567	56.6
Type of Area		
Urban	722	72.1
Rural	280	27.9
Knowledge about use of AB and AMR		
As soon as I feel better, I will stop taking AB	198	19.8
I heard of the term AMR	634	63.3
heard of the term AMR from doctor	209	20.9
The use of AB in agriculture has an impact on the success of treatment in humans	325	32.4
Many bacteria are becoming increasingly resistant to AB	824	82.2
If a bacterium is resistant, it becomes very difficult or almost impossible to treat the infection it causes	792	79.0
AMR is a problem that can affect me or my family	729	72.8
AMR is a problem in other countries, but not here	324	32.3
AMR is a problem only for frequent users of AB	610	60.9
Resistant bacteria can be transmitted from person to person	564	56.3
AMR infections can reduce the success of medical procedures	739	73.8
Attitudes about use of AB and AMR		
MDs prescribe AB only when there is an indication for it	855	85.3
People should be treated with AB only when a doctor prescribes them	931	92.9
Animals should be treated with AB only when prescribed by a veterinarian	860	85.8
Parents should be sure that children’s vaccinations are carried out regularly	929	92.7
People should wash their hands regularly	974	97.2
AMR happens when your body becomes resistant to AB	800	79.8
Pharmaceutical companies are constantly creating new AB	697	69.6

M—male; F—female; U—urban; R—rural; AB—antibiotics; AMR—antimicrobial resistance.

**Table 2:** Practice about the use of AB according to age, gender, and area.

Attitudes about the Use of AB	Age, *n* (%)		*p*	Gender, *n* (%)		*p*	Place of Residence, *n* (%)		*p*	Total *n* (%)
	16–44	45–72		M	F		U	R		
Last time I used AB										
In the previous month	84 (14.7)	82 (19.1)	**0.008**	62 (14.3)	104 (18.3)	**0.001**	115 (15.9)	51 (18.2)	0.256	166 (16.6)
In the last 2 to 6 months	178 (31.1)	92 (21.4)		104 (23.9)	166 (29.3)		199 (27.6)	71 (25.4)		270 (26.9)
In the last 7 to 12 months	71 (12.4)	74 (17.2)		63 (14.5)	82 (14.5)		98 (13.6)	47 (16.8)		145 (14.5)
More than a year ago	144 (25.1)	105 (24.5)		104 (23.9)	145 (25.6)		189 (26.2)	60 (21.4)		249 (24.9)
Never	20 (3.5)	15 (3.5)		24 (5.5)	11 (1.9)		28 (3.9)	7 (2.5)		35 (3.5)
I cannot remember	76 (13.3)	61 (14.2)		78 (17.9)	59 (10.4)		93 (12.9)	44 (15.7)		137 (13.7)
AB treatment in the last year										
Never	204 (35.6)	154 (35.9)	0.176	175 (40.2)	183 (35.7)	0.067	265 (36.7)	93 (33.2)	0.182	358 (35.8)
Once	202 (35.3)	138 (32.2)		133 (30.6)	207 (36.5)		251 (34.8)	89 (31.8)		340 (33.9)
Twice	83 (14.5)	83 (19.3)		73 (16.8)	93 (16.4)		111 (15.4)	55 (19.6)		166 (16.6)
Three times	38 (6.6)	30 (7.0)		24 (5.5)	44 (7.8)		43 (6.0)	25 (8.9)		68 (6.8)
I cannot remember	46 (8.0)	24 (5.6)		30 (6.9)	40 (7.1)		52 (7.2)	18 (6.4)		70 (7.0)
The number of used AB										
Only 1 AB	418 (72.9)	274 (63.9)	**0.006**	301 (69.2)	391 (69.0)	0.147	511 (70.8)	181 (64.6)	0.114	692 (69.1)
Combination of 2 AB	107 (18.7)	96 (22.4)		82 (18.9)	121 (21.3)		145 (20.1)	58 (20.7)		203 (20.3)
Combination of 3 or more AB	28 (3.9)	30 (7.1)		23 (5.3)	35 (6.2)		37 (5.1)	21 (7.5)		58 (5.8)
I cannot remember	20 (3.5)	29 (6.8)		29 (6.7)	20 (3.5)		29 (4.0)	20 (7.1)		49 (4.9)
During the last use AB were prescribed by the doctor	480 (83.8)	365 (85.1)	0.511	345 (79.3)	500 (88.2)	**0.001**	619 (85.7)	226 (84.3)	0.131	845 (84.3)

M—male; F—female; U—urban; R—rural; AB—antibiotics; *p* values < 0.05 are bolded (Chi square test).

**Table 3:** Knowledge about the use of AB and AMR according to age, gender, and area.

Knowledge about the Use of AB	Age, Years *n* (%)		*p*	Gender, *n* (%)		*p*	Place of Residence, *n* (%)		*p*
	16–44	45–72		M	F		U	R	
As soon as I feel better, I will stop taking AB	119 (27.4)	79 (18.4)	0.212	279 (64.1)	441 (77.8)	**<0.001**	128 (17.7)	70 (25.0)	**0.001**
I heard of the term AMR	404 (70.5)	230 (53.6)	**<0.001**	235 (54.0)	399 (70.4)	**<0.001**	490 (67.9)	144 (51.4)	**<0.001**
I heard of the term AMR from doctor	130 (22.7)	79 (18.4)	**0.010**	67 (15.4)	142 (25.0)	**<0.001**	174 (24.1)	35 (12.5)	**<0.001**
The use of AB in agriculture has an impact on the success of treatment in humans	173 (30.2)	152 (35.4)	0.080	119 (27.4)	206 (36.3)	**0.006**	235 (32.5)	90 (32.1)	0.890
AMR is a problem only for those people who use AB frequently	320 (55.8)	290 (67.6)	**<0.001**	268 (61.6)	342 (60.3)	0.678	424 (58.7)	186 (66.4)	**0.025**
Many bacteria are becoming increasingly resistant to AB	478 (83.4)	346 (80.7)	0.257	334 (76.8)	490 (86.4)	**<0.001**	615 (85.2)	209 (74.6)	**<0.001**
If a bacterium is AMR, it becomes very difficult or almost impossible to treat the infection it causes	459 (80.1)	333 (77.6)	0.257	316 (72.6)	476 (84.0)	**<0.001**	579 (80.2)	213 (76.1)	0.150
AMR is a problem that can affect me or my family	429 (74.9)	300 (69.9)	0.082	298 (68.5)	431 (76.0)	**0.008**	546 (75.6)	183 (65.4)	**0.001**
AMR is a problem in other countries, but not here	163 (28.4)	161 (37.5)	**0.002**	158 (36.3)	166 (29.3)	**0.018**	212 (29.4)	112 (40.0)	**0.001**
AMR bacteria can be transmitted from person to person	318 (55.6)	246 (57.3)	0.581	230 (53.0)	334 (58.9)	0.062	418 (58.0)	146 (52.1)	0.095
AMR infections can reduce the success of medical procedures	423 (73.8)	316 (73.7)	0.954	302 (69.4)	437 (77.1)	**0.006**	547 (75.8)	192 (68.6)	**0.020**

M—male; F –female; U—urban; R—rural; AB—antibiotics; AMR—antimicrobial resistance; *p* values < 0.05 are bolded (Chi square test).

**Table 4:** Attitudes about the use of AB and AMR according to age, gender, and area.

Attitudes about the Use of AB	Age, Years *n* (%)		*p*	Gender, *n* (%)		*p*	Place of Residence, *n* (%)		*p*
	16–44	45–72		M	F		U	R	
People should be treated with AB only when a doctor prescribes them	530 (92.5)	401 (93.5)	0.667	390 (89.7)	541 (95.4)	**0.001**	687 (95.2)	344 (87.1)	**<0.001**
Animals should be treated with AB only when prescribed by a veterinarian	497 (86.7)	363 (84.6)	0.443	341 (78.4)	519 (91.5)	**<0.001**	636 (88.1)	224 (80.0)	**0.004**
Parents should be sure that children’s vaccinations are carried out regularly	527 (92.0)	402 (93.7)	0.579	392 (90.1)	537 (94.7)	**0.015**	678 (93.9)	251 (89.6)	**0.046**
People should wash their hands regularly	555 (96.9)	419 (97.7)	0.732	414 (95.2)	560 (98.8)	**0.003**	702 (97.2)	272 (97.1)	0.873
Doctors should prescribe AB only when there is an indication for it	483 (84.3)	372 (86.7)	0.075	361 (83.0)	494 (87.1)	0.120	612 (84.8)	243 (86.8)	0.484
AMR happens when your body becomes resistant to AB	457 (79.8)	343 (80.0)	0.938	329 (75.6)	471 (83.1)	**0.004**	593 (82.1)	207 (73.9)	**0.004**
Pharmaceutical companies are constantly creating new AB	389 (67.9)	308 (71.8)	0.316	294 (67.6)	403 (71.1)	0.486	519 (71.9)	178 (63.6)	**0.022**

M—male; F—female; U—urban; R—rural; AB—antibiotics; AMR—antimicrobial resistance; *p* values < 0.05 are bolded (Chi square test).

**Table 5:** The complete list of risk factors and their association with AB use in practice in the general population of Gornje Podrinje.

Independent Variables	B	SE	β	*p*	Lower 95% CI for B	Upper 95% CI for B
Constant	33.475	6.399		<0.001	20.918	46.031
Age	−0.262	0.948	−0.008	0.782	−2.122	1.598
Gender	3.886	1.898	0.063	0.041	0.162	7.611
Place of residence	−1.254	2.115	−0.018	0.554	−5.405	2.897
Knowledge about AB use and AMR	0.393	0.055	0.226	<0.001	0.285	0.501
Attitudes about AB use and AMR	0.338	0.062	0.170	<0.001	0.217	0.460

AB—antibiotics, AMR—antimicrobial resistance, B—unstandardized coefficients B, SE—standard error, β—standardized Beta coefficients, CI—confidence interval.

## Data Availability

The data underlying the results presented in the study are available from office-mf@ues.rs.ba.
